# Should an Institution That Has Commercial Rights in a New Drug or Device Be Allowed to Evaluate the Technology?

**DOI:** 10.1371/journal.pmed.0020009

**Published:** 2005-01-25

**Authors:** Ross McKinney, David Korn

## Abstract

Background to the debate: In the United States, the passage of the Bayh–Dole Act in 1980 encouraged universities to license inventions for commercial development. Although this financial incentive can stimulate academic researchers to discover new drugs and devices, there is concern that the possibility of monetary reward could distort investigators' objectivity.

## Abstract 2

In the US, universities are encouraged to license inventions for commercial development. Although this financial incentive can stimulate academics to discover new treatments, it might also distort the investigators' objectivity

## Ross McKinney's Viewpoint: Universities Should Be Allowed, Provided the Trial Is Approved by an External Review Board

One of the principal missions of an academic health center is to advance the understanding and treatment of disease through clinical research. In this pursuit, there is a need for checks and balances. When Jesse Gelsinger, a relatively healthy young adult, died in Philadelphia during a clinical trial of a novel adenovirus-based genetic therapy for ornithine transcarbamylase deficiency, it was a tragedy [1]. In retrospect, there were many clues that there were problems with the adenovirus vector, clues that neither the investigator nor the institution pursued.

Attorney Alan Milstein made the case that the investigator and institution were both blinded to these problems by their heavy financial investment in the technology, an investment worth millions of dollars [2]. Though the legal case was settled out of court, it created a de facto standard that institutions with commercial rights in a new drug or technology should not be allowed to pursue clinical trials involving that new technology. I do not believe that such a blanket prohibition is necessary.

At its core, the issue revolves around conflicts of interest. In clinical research, the investigator should be primarily an advocate for the patient or volunteer. The core reason to perform clinical research is to create generalizable knowledge about a therapy, patient population, or a disease process with the long-term intent of improving human health. The interests of the patient and investigator should be fully aligned. However, most physicians in clinical research have other, more personal motivations, intermixed with the desire for progress. Successful research projects can lead to publications, promotions, grant renewals, and per case clinical trial enrollment fees. Some investigators have intellectual property rights that may have very substantial financial value if the drug or device reaches the level of approval by the United States Food and Drug Administration. These investigators stand to gain personally if the clinical trial is successful, a situation that has the potential to distort the investigator's objectivity, and may lead to a less honest relationship with study volunteers.[Fig pmed-0020009-g001]

**Figure pmed-0020009-g001:**
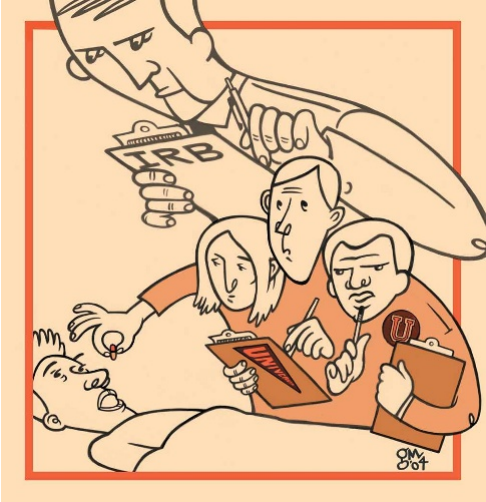
An external IRB could provide independent oversight of the trial (Illustration: Giovanni Maki)

In order to ensure that investigators are honest with potential research volunteers, the system of institutional review boards (IRBs) evolved. The IRB approves the informed consent document, which should describe the clinical experiment in a clear and dispassionate way to patients and their families. IRBs are largely made up of faculty and staff from the institution, although there are also public members and nonscientists on most IRB panels. The IRB must remain autonomous and be able to hold up or stop an investigation. There is an obligation that the IRB first and foremost think about patient rights and safety.

The passage of the Bayh–Dole Act in 1980 enabled universities to license inventions for commercial development [3]. The closer to Food and Drug Administration approval the drug or technology is at the time of licensure, the more valuable it becomes. Therefore, universities have an incentive to advance the clinical development of inventions by their faculty. In this regard, they are very much like corporate sponsors of research, subject to the same Food and Drug Administration oversight as corporations.

In terms of performing clinical trials using new technologies in which it has a financial interest, how is a university different from a corporate sponsor? In regard to patient safety, one primary distinction rests with the IRB. The corporate sponsor will present the research protocol to an independent commercial IRB, the university to its own IRB. Yet in both cases, there are potential conflicts of interest. The university IRB members will have a conflict of interest between the investments of their employer and the rights of the research volunteers. Independent commercial IRBs depend on pleasing corporate customers for their continued existence, and there is an unstated expectation that they will both be fast and produce rulings consistent with corporate expectations (which in most cases include a desire to do the research ethically).[Fig pmed-0020009-g002]

**Figure pmed-0020009-g002:**
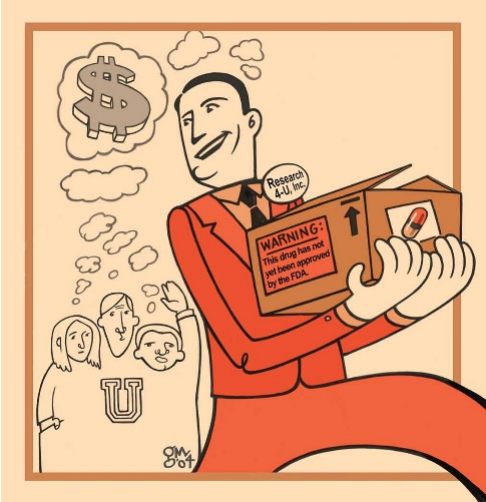
A university's financial conflicts could influence the conduct of the trial (Illustration: Giovanni Maki)

At the university level a logical and conservative solution to the problem of institutional conflicts is to require that an IRB from outside the institution become the IRB of record when such a conflict arises. This external IRB could be either an independent commercial IRB or one of another university. The key is to grant the IRB independence and the authority to provide real oversight.

There are other elements of conflicts of interest that need to be considered when the institution has a commercial interest, but most have more to do with the management of personal conflicts than institutional conflicts. The institution needs to assure the presence of an independent data safety monitoring board, thorough audits of good clinical practice, and a publications committee that will ensure submission of all meaningful study results, whether positive, negative, or neutral. Anyone subjectively evaluating patient data should be as free of conflicts as possible. These steps can be formulaically required, which should allow for performance of clinical research despite the presence of institutional commercial interests.

## David Korn's Viewpoint: Academic Biomedical Research Must Be Free from the Taint of Financial Compromise

United States research universities, and especially their academic medical centers, have greatly benefited from their uniquely privileged status in our society. That status is rooted in public confidence and trust that these institutions and their faculties will be independent and impartial in fulfilling both their academic mission to create, transmit, and preserve knowledge, and their duty to the general society to serve as credible, trustworthy arbiters of knowledge. One important mark of this status has been the remarkably consistent generosity of public support for biomedical research. Another has been the noteworthy deference of the federal government to university autonomy, and the light hand with which the sponsoring agencies historically have overseen the conduct of university research.

When federal interposition occurred, it typically responded to widely publicized episodes of research misconduct, sometimes intertwined with egregious financial self interests of investigators; these episodes legitimately questioned the effectiveness of institutional oversight. Nevertheless, regulations consistently focused more on defining the metes and bounds of the permissible than on prescriptive mandates, and their implementation was effected largely through the mechanism of “assurances”—commitments that institutions would faithfully safeguard the specified perimeters of acceptable conduct.

Awardee institutions thus bear primary responsibility for assuring the credibility and integrity of federally sponsored research. Public confidence in the trustworthiness of these institutions is critical, and yet nowhere is it more fragile than in biomedical research involving human participants. That confidence eroded in the 1980s and 1990s because of reports of scientific misconduct and of individual and institutional financial self interests in clinical trials. Scathing reports from federal oversight agencies and angry congressional hearings questioned whether financially self-interested institutions could any longer be trusted to guard the welfare of research participants or the integrity of clinical research.

In 2001, the Association of American Medical Colleges convened a task force to examine and make recommendations on individual and institutional financial self interests in clinical research. The task force began by recognizing four important trends over the past three decades. First, the nature and culture of academic biomedical research have changed, bringing the potential of commercial relevance even to the most fundamental of scientific discoveries. Second, there has been enormous growth in the extent and depth of interactions between research universities and industry, especially in biomedicine. Third, the public has become increasingly impatient that its extraordinary investments in research yield more effective disease preventions and therapies. Fourth, the involvement of academic researchers in the translation of their discoveries has been essential in bringing those discoveries to market and to the benefit of public health.

But the task force, in its two reports, asserted that both individual and institutional financial conflicts of interest in clinical research could be problematic [4,5]. It recommended urgent and substantial refinement and strengthening of institutional policies and practices for monitoring, managing, and—when necessary—extinguishing such conflicts.

Both reports rest on a common set of core principles. The most important is that institutions should regard all significant financial interests in research involving human participants as potentially problematic. Where such interests exist, there should be a rebuttable presumption that the concerned individual or institution should not conduct the research, absent compelling circumstances. Importantly, the task force, after intense debate, rejected categorical prohibitions lest they unintentionally impede the translation of research discoveries into tangible public benefits.

The task force acknowledged that the issue of institutional financial self interests is extraordinarily complex and sensitive, since it touches the very core of institutional autonomy. But the fact that an institution has a financial interest per se should raise a strong presumption against its participation in the clinical testing of that product. Public accountability and scientific integrity require that all research results emanating from academic medicine be as free as possible from the taint of financial compromise. Adding human participants to the research mix should raise the barrier to the highest level and require compelling justification for any participation by a financially self-interested institution.

The task force did not define “compelling,” believing that each institution should make that determination based on disinterested scrutiny of the facts and circumstances of each case. For example, there may exist in a given institution a unique capability, without which the proposed research involving human participants could not be conducted as effectively or safely, or at all. In these instances, the public and science deserve access to that capability, provided the necessary safeguards are put in place to mediate the conflicting interests. In all such instances, protection of scientific integrity and the welfare of research participants must remain the foremost priority of both investigator and institution.

This narrow window avoids absolute prohibition while striving to prevent institutional participation where credible alternatives exist. Only by such stringent self-policing can we sustain the trustworthiness and credibility of biomedical research, researchers, and their institutions, while continuing vigorously to promote the translation of biomedical discovery for the public's benefit.

## Ross McKinney's Response to David Korn's Viewpoint

The public has every right to expect that academic institutions are working first for the public's interest. This value is even codified in the laws granting these institutions tax-exempt status. The public also expects that new, more effective therapies will be developed swiftly as a consequence of its support for academic research.

The inventor of a new technology is always more motivated to see it through to widespread use than anyone else. This motivation, which may be as simple and benign as curiosity or as easy to understand as a financial incentive, is a powerful force driving human research. This force can be disciplined and controlled by the IRB and policies on conflicts of interest. Personal investment in research is, nevertheless, an important driver of scientific progress.

When society makes an unnecessarily broad assumption that nearly all research with financial implications for investigators or their institution is potentially corrupted, a brake is placed on progress. Society will be better served by establishing clear guidelines and formalizing oversight of the research process than by rigidly limiting clinical research affected by conflict of interest. As examples, clinical trials should have independent data safety monitoring boards charged to review the study design, execution, data analysis, and publication of results. The IRB system should be strengthened in its independence through the use of community members. And, to be certain that institutional conflict of interest is avoided, an IRB from outside the institution in most cases will be preferable

Society wants better treatments. The fact that an inventor has mixed motives for developing a new treatment has always been acknowledged. The need to carefully manage the experimental process in human studies has always been understood. However, when rules minimize the role of inventors at academic centers, by forcing trials of their new ideas to go to outside institutions, society loses more than it gains. The incremental gain in safety is likely to be small (particularly if oversight is well established), while the decrease in speed of development will be significant.

## David Korn's Response to Ross McKinney's Viewpoint

There are many similarities between the position espoused by Ross McKinney and my position. Most saliently, we both recognize the critically important role played by academic biomedical scientists in making discoveries and in facilitating their efficient translation into beneficial products. Neither of us proposes that academic investigators, or their institutions, should be flatly prohibited from trying to foster that translation in the presence of financial self interests.

But there is an important difference. McKinney's approach for dealing with institutional conflicts of interest depends critically on the engagement of external agents to monitor closely both scientific integrity and the welfare of human participants. That, in my view, would require such deep interposition of those agents into the conduct of academic research as to be not only unprecedented but unfeasible. Beyond that, the approach falls short with respect to the maintenance of institutional trustworthiness and protection of public trust.

Routine clinical assessment of technologies by financially interested institutions fosters public cynicism and distrust of the motives of academic biomedical researchers. The protective mechanisms recommended by McKinney are opaque to the public and reflect a “business as usual” image that fails fully to account for the markedly changed circumstances and perceptions of academic biomedical research. Most important, the mechanisms appear to be aimed primarily at protecting institutions' financial interests.

By contrast, the American Association of Medical Colleges formulation urges that any institutional involvement in clinical research involving human participants in the presence of financial conflicts must be predicated on the presence or absence within the institution of demonstrably unique capability. This approach offers a much higher and more credible standard that aims to protect not only participant well-being and scientific integrity, but also institutional trustworthiness and public trust.

## Abbreviation

IRB: institutional review board
